# Context-specific temporal learning with non-conflict stimuli: proof-of-principle for a learning account of context-specific proportion congruent effects

**DOI:** 10.3389/fpsyg.2014.01241

**Published:** 2014-10-30

**Authors:** James R. Schmidt, Céline Lemercier, Jan De Houwer

**Affiliations:** ^1^Department of Experimental Clinical and Health Psychology, Ghent University, GhentBelgium; ^2^Laboratoire Travail et Cognition, Université de Toulouse, ToulouseFrance

**Keywords:** context, temporal learning, proportion congruency, conflict adaptation, cognitive control, attention, contingency learning, contrast

## Abstract

The conflict adaptation account proposes that participants adjust attention to target and distracting stimuli in response to conflict. This is argued to explain the proportion congruent effect, wherein the congruency effect decreases as the proportion of conflicting incongruent trials increases. Some reports further argue that this conflict adaptation process can be context-specific. This paper presents a proof-of-principle for a competing account. It is suggested that such context-specific effects might be driven by very basic temporal learning processes. In the reported experiment, we manipulated stimulus contrast in place of congruency. In one location, stimulus letters were mostly easy to identify (high stimulus contrast). In the other location, letters were mostly hard to identify (low stimulus contrast). Participants produced a larger contrast effect in the mostly easy context. Along with supplemental analyses investigating the role of context switching and previous trial response times, the results are consistent with the notion that different rhythms of responding are learned for an easy versus hard location context. These results suggest that context-specific proportion congruency effects might result, in whole or in part, from temporal learning. Conflict adaptation may or may not play an additional role.

## INTRODUCTION

Learning about *when* to respond is arguably as important as learning *what* to respond when interacting with the environment. Whether for determining the causal relation between events or using said causal knowledge to optimally respond to stimuli in the fastest and most accurate manner possible, both contingency and temporal information are critical for successful performance. In the context of psychological experiments, detecting regularities allows for the anticipation of future events on subsequent trials, thus benefiting performance when expectations match reality. For instance, when the series of responses in a task follow a predictable order, responses are sped up relative to a random ordering of responses (Nissen and Bullemer, 1987). Similarly, if a neutral distracting stimulus is predictive of the likely target stimulus, performance is aided when the expected stimulus is presented (e.g., Miller, 1987; Schmidt et al., 2007). Also with timing information, if a cue indicates the likely time at which a stimulus will appear, performance is sped up if the stimulus appears at the expected time (e.g., Hsu et al., 2013).

The learning of contingent and temporal regularity occurs quite easily and has near immediate effects on behavior. One somewhat unfortunate consequence of this fact is that many experiments aimed at investigating something else entirely might end up unintentionally biased by such learning confounds when a regularity exists in the task structure that is learnable by participants. Of particular interest to the present article, much debate has focused on the presence of such learning biases in the cognitive control literature (for a recent review, see Schmidt, 2013a). This paper has two main goals. The more general goal is to investigate the potential role of contextual information in moderating learning, particularly temporal learning. The more specific goal is to discuss how context-specificity in temporal learning relates to an interesting finding in the cognitive control literature, namely, the context-specific proportion congruent (PC) effect. We begin by providing a background for the latter of these two goals, and will then return to the former.

When given the goal to selectively attend to one stimulus while simultaneously ignoring another distracting stimulus, participants are not completely successful at doing so. For instance, in the Stroop task (Stroop, 1935) participants see color words printed in colors. On a *congruent trial*, the word and color match (e.g., the word “blue” printed in blue); whereas on an *incongruent trial*, the word and color mismatch (e.g., “blue” in red). Responses to incongruent trials are slower and less accurate than congruent trials. This congruency effect indicates that the distracting word has partially slipped through the attentional filter, producing conflict.

In the cognitive control literature, it is often assumed that the attentional system adapts to conflict by adjusting the allocation of attentional resources away from the source of conflict (e.g., the distracting word) and/or toward the target stimulus (e.g., the color). This is called the *conflict adaptation account*. One piece of evidence argued to support the conflict adaptation account is the *PC effect*: the greater the proportion of incongruent trials, the smaller the congruency effect (Lowe and Mitterer, 1982). According to the conflict adaptation account (e.g., Botvinick et al., 2001), this occurs because participants detect frequent conflict and adjust attention away from the word.

However, others have argued that the cognitive system might not be reactive to conflict. Instead, findings such as the PC effect might be driven by regularities in the task structure that allow for learning biases (e.g., Schmidt and Besner, 2008; Schmidt, 2013b,c; for a review, see Schmidt, 2013a). For instance, Schmidt and Besner (2008) (see also Mordkoff, 1996; Jacoby et al., 2003; Schmidt, 2013c) argue that such an effect may be largely explained by simple word–color contingency learning. When most of the trials are congruent, then each word is presented most often in its congruent color. The word is therefore a valid cue of the response on congruent trials, and invalid on incongruent trials, thereby increasing the congruency effect. When most of the trials are incongruent, the bias is eliminated or even reversed (depending on the specific manipulation). Thus, the PC effect might be driven by contingency biases, rather than conflict adaptation.

The contingency account often seems to undermine the conflict adaptation account in explaining both behavioral (e.g., Atalay and Misirlisoy, 2012; Schmidt, 2013c) and brain data (Grandjean et al., 2013). However, there are still several findings that may not seem to fit well with the simple learning view. One such finding is the context-specific proportion congruent (CSPC) effect. Corballis and Gratton (2003) used a flanker task in which distracting letters were mostly congruent with the target letter on one side of the screen (e.g., left), but mostly incongruent on the other side of the screen (e.g., right). The congruency effect was larger in the mostly congruent relative to mostly incongruent location. This was initially argued as evidence for hemispheric-specificity in conflict adaptation. However, Crump et al. (2006) replicated this effect in a Stroop-like procedure with *up* and *down* locations, rather than left and right, and argued instead that participants simply adapt to conflict differently in different contextual locations (see also, Wendt et al., 2008). That is, in the location with mostly incongruent trials attention to the word is reduced relative to the location with mostly congruent trials. Similarly, Bugg et al. (2008) presented color–word Stroop stimuli in two fonts. With one font, the word was mostly congruent; and with the other, the word was mostly incongruent. The congruency effect was larger for the mostly congruent font. This again might suggest that participants can dynamically (i.e., on a trial-to-trial basis) adjust their attention to distracting words on the basis of contextual cues, such as fonts or locations.

What is interesting about CSPC effects is that they have been argued to be difficult to explain via a contingency learning account. The distracting word, for instance, is not predictive of which response to make. Indeed, every word is both mostly congruent *and* mostly incongruent, depending on the context. Similarly, the context cue (e.g., location) is not predictive of what response to make. Thus, such data have been taken as strong support for the notion that conflict adaptation does occur. However, the CSPC effect could be explained with a contingency learning mechanism by assuming that multiple irrelevant cues are combined to predict the response. For instance, in the mostly congruent location “blue” predicts a blue response, whereas in the mostly incongruent location “blue” predicts a green response. Thus, it is not unreasonable to assume that participants might combine two distracting cues, such as the word and stimulus location, to jointly predict the response. Indeed, Mordkoff and Halterman (2008) demonstrated exactly this in three flanker experiments. Color and shape combinations were used as flankers to unrelated target stimuli. Just like in a CSPC experiment, each flanker color was predictive of one response (e.g., left key press) when presented with one shape, and the other response (e.g., right) when presented with a different shape. Participants responded faster for the high contingency color–shape conjunctions than the low-contingency color–shape conjunctions. More generally, work on occasion setting suggests that participants can use conjunctive stimulus information quite readily (for a review, see Holland, 1992).

However, even if contingency learning can be context-specific in this way, there are still results that are not explainable by such a mechanism. Of particular interest, Crump and Milliken (2009; see also Heinemann et al., 2009; Reuss et al., 2014) used both context and transfer items in a single CSPC experiment. *Context items* were manipulated for PC across locations. *Transfer items* were not manipulated for PC, having equal congruency proportions in both locations. While combining the word and the location might provide predictive information on context items, this would be impossible for transfer items. A CSPC effect was observed for *both* item types, however. A contingency learning account is therefore unsatisfactory for explaining the effects on transfer items, because such items were frequency-unbiased. Indeed, the fact that an effect occurs for the transfer items at all indicates that behavior is being influenced, at least in part, in a non-item-specific way. That is, transfer items only produce a CSPC effect due to the influence of the intermixed context items. Contingency biases likely do play some role in producing the CSPC effect, but it is clear that they are not the whole story.

However, another learning bias that might explain a component of CSPC effects, particularly for frequency-unbiased transfer items, is temporal learning. In addition to learning what response to make to a stimulus, participants can also learn the timing of responses (Matzel et al., 1988; Taatgen and Van Rijn, 2011). According to the *temporal learning account*, participants learn about *when* to respond based on the rhythm of the task (e.g., Grosjean et al., 2001). This timing information can influence subsequent behavior. For instance, the speed of responding to one task affects the speed of responding to a second, intermixed task (for a review, see Los, 1996). Note that such effects are by definition not (entirely) item-specific: performance on some items affects performance on others. Multiple potential mechanisms for such effects have been proposed. For instance, temporal information might be used to alter response caution (Van Maanen et al., 2011), to balance speed and accuracy (Kinoshita and Mozer, 2006), or to make time-criterion adjustments (Lupker et al., 1997).

There are multiple possible mechanisms for temporal learning. Most can explain components of the PC effect. For instance, Schmidt (2013b) suggested that participants develop expectancies of when they will be able to respond on the basis of previous trials. When a response is sufficiently active at the expected time, a shortcut in responding can occur. In the mostly congruent condition, the expected time to respond will be relatively early in the trial, due to the preponderance of congruent trials. Subsequent congruent trials will thus benefit from this temporal expectancy, because a response is likely to be active enough at the expected time, allowing a response to be produced even faster than usual (e.g., because the response threshold is temporally decreased at the expected time). In contrast, incongruent trials will not benefit, because there will be insufficient evidence for the correct response at the expected time, meaning that evidence has to continue accruing and the expectancy window is missed. This produces a relatively large congruency effect. In the mostly incongruent condition, the expected time to respond will be relatively later in the trial, due to the preponderance of incongruent trials. Subsequent incongruent trials will thus benefit from this temporal expectancy, because evidence for the correct response will be strong enough at the expected time to benefit from an expectancy-based shortcutting in responding. In contrast, congruent trials will not benefit, because a response will have already been made before the expected time to respond, thus missing out on the added benefit of matching the rhythm of previous trials. This produces a relatively smaller congruency effect.

According to the temporal learning account, the PC effect is not due to conflict *per se*, but merely to the speed of responding in the task. In support of this, large portions of the PC effect are driven by the speed of responding to previous trials (Kinoshita et al., 2011; Schmidt, 2013b). Moreover, Schmidt (2013b) showed that a “pseudo” PC effect can be produced without manipulating conflict and without the presence of a distracting stimulus. Instead, target letters were manipulated for stimulus contrast, with high contrast (easy to see) and low contrast (hard to see) letters. Thus, fast (high contrast) and slow (low contrast) responses are still made, but without a conflict manipulation. To manipulate timing, letters were presented most often in high contrast (mostly easy) for half of the participants and most often in low contrast (mostly hard) for the other half. The contrast effect (i.e., low minus high contrast trials) was larger in the mostly easy relative to the mostly hard condition. This *proportion easy effect* therefore parallels the PC effect. It was further demonstrated by Schmidt (2014) that the proportion easy effect is *not* (at least primarily) item-specific. Some context letters were manipulated for “proportion easy” whereas other intermixed transfer items were not. While there was some (non-significant) hint that the proportion easy effect might have been larger for context items, a reliable proportion easy effect was observed for both item types. This indicates that participants can learn the overall speed of the task, which then produces larger effects (of whatever sort) in the mostly easy context relative to the mostly hard context, even for frequency-unbiased transfer items.

It might then be suggested that participants in the experiment of Crump and Milliken (2009) produced larger congruency effects in the mostly congruent location because of a different temporal expectancy for each location. In other words, temporal learning might be context-specific. The expectancy for a relatively quick response for stimuli presented in the mostly congruent location (i.e., due to a preponderance of fast congruent trials) benefits congruent trials, whereas the expectancy for a relatively slow response for stimuli presented in the mostly incongruent location (i.e., due to a preponderance of slow incongruent trials) benefits incongruent trials. It is, of course, extremely difficult to de-confound temporal learning and conflict biases in an experiment, because proportion congruency and the proportion of fast responses are inherently confounded. Thus, it is difficult to conceive a way to test for conflict adaptation effects independent of temporal biases. It is possible, however, to provide a proof-of-principle that temporal learning biases can produce an interaction that mimics the CSPC effect, even in the absence of conflict in the task. In that vein, the current experiment aims to test the context-specific temporal learning account by manipulating the proportion of easy (high contrast) items across two display locations. If the contrast effect is observed to be larger in the mostly easy location than the mostly hard location, then this would be consistent with the notion that temporal learning can be context-specific.

Such a finding would also provide an important proof-of-principle that the context-specific temporal learning account might provide a viable alternative interpretation of the CSPC effect. Because there is no *conflict* in the task (indeed, there are no distracters at all), the conflict adaptation account would not predict an effect. The temporal learning account, on the other hand, merely assumes that the difference in response time (RT) between easy and hard items is influenced by the proportion of easy and hard items. In other words, it is important that participants respond faster in one context than the other, but it is not important *why* participants respond faster or slower in a given context. Thus, an experiment constructed analogously to a CSPC task but without a manipulation of conflict, should nevertheless produce the same interaction.

In the interest of better understanding context-specificity in temporal learning, the experiment tested two other side questions. First, it seemed possible that the proportion easy effect might be dependent in some way on whether the context of the previous trial matched the context of the current trial. For instance, it might be the case that a context-specific proportion easy effect is only observed on context (i.e., location) repetition trials, where the context remains consistent from one trial to the next. On context alternation trials, it might be the case that there is a cost of shifting contexts that results in the elimination (or reduction) of temporal expectancies. Evidence consistent with this notion is present in CSPC studies (King et al., 2012a,b).

Second, it is known that the RT of the immediately preceding trial has a large influence on performance on the next trial. For instance, not only is previous trial RT highly correlated with current trial RT, but congruency effects increase the faster the previous trial RT (e.g., Kinoshita et al., 2011; Schmidt, 2013b). This is highly consistent with time-based learning accounts. In these accounts, participants learn *when* to respond on the basis of previous performance, so it only stands to reason that the immediately-preceding trial should have some measurable impact on current trial performance. This follows the same logic already discussed when explaining the temporal learning account. For instance, following a fast response to an easy item, another easy item should be able to be responded to particularly fast (just like a fast rhythm of responding should benefit responding more on congruent than incongruent trials). Following a slow response to a hard item, another hard item should be responded to faster than typical (just like a slow rhythm of responding should benefit responding more on incongruent than on congruent trials). Because the temporal learning account is proposed to explain the context-specific proportion easy effect, a few predictions follow. First but least important, previous and current RT should correlate. Second, the contrast effect should be larger the faster the previous RT. Again, this is because easy trials will benefit most following a fast (easy) trial, whereas hard trials will not, resulting in a large contrast effect following fast responses. The exact opposite is true following a slow (hard) trial, resulting in smaller contrast effects following slower RTs. Third, the impact of previous trial RT should be especially large when the context repeats from one trial to the next. When the context alternates, the previous RT does not correspond to the same context, so the effect should be attenuated. We therefore conducted analyses that were specifically designed to assess these predictions. Notably, it is unclear why the conflict adaptation or any other attentional filtering account should make any of these predictions regarding previous RT. First, there is no conflict in the task to adapt to. Second, these accounts merely argue that attention is adapted to the conflict level associated with a given context, not to time information.

## MATERIALS AND METHODS

### PARTICIPANTS

Participants were 60 Ghent University undergraduates who participated in exchange for €5. The research was approved by the Ethical Committee at Ghent University. Participants provided informed consent before participating.

### APPARATUS

Stimulus and response timing were controlled by E-Prime 2 (Psychology Software Tools, Sharpsburg, PA, USA). Participants responded to the letters D, F, J, and K with the D, F, J, and K keys of an AZERTY keyboard of a laptop PC, respectively. The laptop PC had a 15″ monitor.

### MATERIALS AND DESIGN

The stimulus letters for the experiment were D, F, J, and K. Letters were presented on a dark gray background (100,100,100). Before the main experiment, there was a 24-trial practice phase, consisting of six presentations of each letter in the center of the screen in white (255, 255, 255). In the main phase of the experiment, letters were presented in either high contrast gray (200, 200, 200) or low contrast gray (110, 110, 110), for a total of eight unique letter–contrast combinations. Letters were presented in bold, 18 pt Courier New font on a 640 × 480 resolution screen setting. On half of the trials, the letter appeared on the top half of the screen (four lines up from the center), and on the other half of the trials on the bottom half of the screen (four lines down from the center). In one location (mostly easy), each letter was presented 70% of the time in high contrast and 30% in low contrast. In the other location (mostly hard), the proportions were reversed. Which location (above or below) served as the mostly easy location was counterbalanced across participants. The contrast effect is defined as the difference between high and low contrast trials. A context-specific proportion easy effect is defined as a larger contrast effect in the mostly easy location context relative to the mostly hard context. For the main part of the experiment, there were a total of 300 trials selected at random with replacement.

### PROCEDURE

Participants were instructed to press the key corresponding to the letter on the screen (e.g., press the K key for the letter K). Each trial began with a blank screen for 500 ms, followed by the target letter for 2000 ms or until a response was made. Correct responses were immediately followed by the next trial, whereas incorrect responses and trials on which participants failed to respond in 2000 ms were followed by a centrally located “XXX” in red (255, 0, 0) for 500 ms.

## RESULTS

Mean correct RTs and percentage errors were calculated. Analyses were conducted with a linear mixed effect (LME) model in order to assess any overall benefits for stimuli presented in one of the two stimulus locations and/or interactions between stimulus location and the other factors. Note that such an analysis cannot easily be performed with a standard repeated-measures ANOVA^1^. For those unfamiliar with LME models, it is sufficient to know that we performed our analysis in a roughly identical fashion to a typical repeated-measures ANOVA, only with a type of dataset that ANOVA cannot handle (see Footnote 1). We performed all analyses using the MIXED command in SPSS with maximum likelihood estimation. Note that while LME *can* be used for much more advanced analyses, we used LME to produce a simple analysis roughly equivalent to repeated-measures ANOVA. For the initial analyzes, the fixed factors were contrast (high vs. low), context (mostly easy vs. mostly hard), stimulus location (above vs. below), and their interactions. High contrast, mostly easy, and below were coded as 1, and the other levels as 0. The mean RT for each participant in each of the unique factor combinations were used for the analysis. Participants were inserted as the single random effect.

### RESPONSE TIMES

The correct RT data are presented in **Table 1**. The data revealed a significant main effect of contrast, *F*_(1,180)_ = 476.422, *p* < .001, $\eta_{p}^{2}$ = 0.89, indicating faster responses to high contrast stimuli. There was also a main effect of context, *F*_(1,180)_ = 13.628, *p* < .001, $\eta_{p}^{2}$ = 0.19, indicating slower overall responses in the mostly easy condition^2^. Critically and as predicted, contrast and context interacted, *F*_(1,180)_ = 4.958, *p* = 0.027, $\eta_{p}^{2}$ = 0.08, indicating a smaller contrast effect in the mostly hard condition. There was also a main effect of stimulus location, *F*_(1,180)_ = 46.182, *p* < 0.001, $\eta_{p}^{2}$ = 0.44, and an interaction between stimulus location and contrast, *F*_(1,180)_ = 29.338, *p* < .001, $\eta_{p}^{2}$ = 0.34. These were due to longer overall RTs and larger contrast effects in the bottom location, respectively. Location did not interact with context, *F*_(1,60)_ = 0.112, *p* = 0.739, $\eta_{p}^{2}$ < 0.01. Finally, the three-way interaction between location, contrast, and context was also not significant, *F*_(1,180)_ = 1.192, *p* = 0.276, $\eta_{p}^{2}$ = 0.02, showing that the context-specific temporal learning effect was roughly equivalent in both locations.

**Table 1:** Experiment response times and errors (SEs in parentheses).

	High		Low		Effect	
	RTs	Errors	RTs	Errors	RTs	Errors
**Above**
Mostly easy	612 (17)	2.8 (0.5)	728 (22)	3.0 (0.5)	116	0.2
Mostly hard	603 (14)	5.6 (0.9)	704 (16)	5.2 (0.6)	101	-0.4
*Difference*					*15*	*0.6*
**Below**
Mostly easy	622 (14)	5.6 (0.5)	824 (19)	5.9 (0.9)	202	0.4
Mostly hard	612 (18)	3.4 (0.6)	770 (20)	3.8 (0.6)	158	0.3
*Difference*					*44*	*0.0*

### PERCENTAGE ERRORS

The error data are also presented in **Table 1**. Generally, the error data were much less sensitive. There was no significant contrast effect, *F*_(1,180)_ = 0.106, *p* = 0.745, $\eta_{p}^{2}$ < 0.01, no context effect, *F*_(1,180)_ = 0.217, *p* = 0.642, $\eta_{p}^{2}$ < 0.01, and no interaction between the two, *F*_(1,180)_ = 0.139, *p* = 0.710, $\eta_{p}^{2}$ < 0.01. The main effect of location was also not significant, *F*_(1,180)_ = 2.222, *p* = 0.138, $\eta_{p}^{2}$ < 0.04. Location and context did interact, *F*_(1,60)_ = 12.723, *p* < 0.001, $\eta_{p}^{2}$ < 0.18. Location and contrast did not interact, *F*_(1,180)_ = 0.425, *p* = 0.515, $\eta_{p}^{2}$ < 0.01. The three-way interaction between location, contrast, and context was also not significant, *F*_(1,180)_ = 0.176, *p* = 0.676, $\eta_{p}^{2}$ < 0.01. Critically, there was no evidence for a speed–accuracy trade-off.

### CONTEXT REPETITIONS

Next, RT data were reassessed for a potential role of repetition versus alternation of the context from one trial to the next. To assess this possibility, we conducted another LME model on correct RTs including the variable context transition (context repetition versus context alternation). Because the previous trial context will be correlated with previous trial contrast (e.g., more high contrast trials if the previous trial was the mostly easy location), we also included the factor of previous trial contrast (high versus low). Thus, we added these two new factors to the LME model, along with their interactions with the other factors. Again, a mean for each unique combination of these factors was computed for each participants for the analysis. In this analysis, all the previously reported results were replicated. Most importantly, the contrast by context interaction remained significant, *F*_(1,898)_ = 9.740, *p* = 0.002, $\eta_{p}^{2}$ = 0.14. Interestingly, this context-specific proportion easy effect was not modulated by context transition, *F*_(1,898)_ = 0.027, *p* = 0.871, $\eta_{p}^{2}$ < 0.01, or by previous trial contrast, *F*_(1,898)_ = 1.667, *p* = 0.197, $\eta_{p}^{2}$ = 0.03. Responses were, however, faster overall if the context repeated, *F*_(1,898)_ = 81.449, *p* < 0.001, $\eta_{p}^{2}$ < 0.58, and the contrast effect was smaller on a context repetition, *F*_(1,898)_ = 4.459, *p* = 0.035, $\eta_{p}^{2}$ < 0.07. There was no main effect of previous trial contrast, *F*_(1,898)_ = 0.021, *p* = 0.886, $\eta_{p}^{2}$ < 0.01, but the contrast effect was larger if the previous trial was high contrast, *F*_(1,898)_ = 6.631, *p* = .010, $\eta_{p}^{2}$ = 0.10. Thus, context transition and previous trial contrast did have an impact on current trial contrast, but not on the critical proportion easy effect. All other effects were non-significant.

### PREVIOUS RESPONSE TIMES

Next, we assessed the possible role of previous trial RTs on the size of the contrast effect. Of course, every trial had a unique previous RT associated with it. Thus, all trials were inserted into the LME. Trials with an error on the current or previous trial were excluded from analyses, however. Participants were again added as a random factor. The fixed main effect factors included the scale variable of previous RT and the binary factors contrast, context, location, previous trial contrast, and context transition. For the binary factors, low contrast, mostly hard, above location, previous low contrast, and context repetition were coded as zero, with the other level of each of these factors codes as one. Previous RT and the RT dependent measure were inverse transformed (–1000/RT, similar to Kinoshita et al., 2011; Schmidt, 2013b) to correct violations of normality,^3^ and were centered on the mean to avoid correlation with the intercept. Investigation of the Q–Q plots revealed no need for trimming the tails of the distribution.

Three models were tested. Model A was the fully factorial model, including all interactions between the six factors. This was obviously a very complex model with far too many terms. We therefore tested two simpler models. Model B was the same as Model A with the exclusion of many non-significant and seemingly irrelevant interactions involving previous RT. Only the main effect of previous RT and its two- and three-way interactions with contrast and context transition were retained. Model C was simpler still. In neither Model A nor Model B was any main effect or interaction involving previous contrast significant. Model C was thus identical to Model B with the exclusion of previous contrast.

To select the best of the three models we assessed the Akaike information criterion (AIC) and the Bayesian information criterion (BIC) scores. For those unfamiliar with such information criteria, AIC and BIC are two different ways to assess the amount of variance explained by a set of factors and can be used to assess whether a more complex model does or does not add anything meaningful to a simpler model. For both AIC and BIC, lower scores indicate a better model. Model A produced the worst (highest) scores of the three models (AIC: 18467; BIC: 18975), and was therefore excluded. Model B produced a slightly better (lower) AIC score than Model C (Model B: 18446; Model C: 18452), but a notably worse BIC score (Model B: 18739; Model C: 18621). The difference between these two measures is not so surprising given the harsher penalty BIC gives to added factors. Whether to favor AIC or BIC scores is a matter of heated contention, but we note that none of the factors that Model C excludes were significant in Model B and the results of the key comparisons were qualitatively the same in both models (i.e., same significant and non-significant effects). We therefore decided to present the simplest model (i.e., Model C).

**Table 2** presents the parameters and statistical tests for Model C. Note that the RT dependent measure was inversed transformed, so the parameter estimates are difficult to relate back to mean RT. However, in the following we will explain what each of these tests show. We first consider the tests *excluding* previous RT (non-shaded cells in **Table 2**). There was a main effect of contrast, indicating overall slower responses to low contrast items. There was also a main effect of context, indicating overall faster responses to mostly hard items. There was no effect of location. There was a main effect of context transition, indicating faster responses for repeated locations. As before, contrast and context interacted, indicating a proportion easy effect. There was also an interaction between location and context, indicating overall slower responses to mostly easy items in the below location. Contrast, context, and location interacted, indicating a larger proportion easy effect in the below location. No other interactions were significant.

**Table 2:** Mixed modeling results.

Variable	Estimate	SE	*t*	*p*
Intercept	-0.071283	0.034188	-2.085	0.041
Contrast	0.415671	0.020591	20.187	<0.001
Context	-0.061880	0.020618	-3.001	0.003
Location	-0.027907	0.048361	-0.577	0.566
Context transition (CT)	-0.109343	0.016075	-6.802	<0.001
Contrast*Context	-0.155539	0.029148	-5.336	<0.001
Contrast*Location	-0.162065	0.028590	-5.669	<0.001
Contrast*CT	-0.020604	0.029425	-0.700	0.484
Context*Location	0.065353	0.029129	2.244	0.025
Context*CT	0.045058	0.029501	1.527	0.127
Location*CT	0.005037	0.022659	0.222	0.824
Contrast*Context*Location	0.248084	0.040793	6.081	<0.001
Contrast*Context*CT	-0.037323	0.041547	-0.898	0.369
Contrast*Location*CT	0.021650	0.040916	-0.529	0.597
Context*Location*CT	-0.065708	0.041111	-1.598	0.110
Contrast*Context*Location*CT	-0.003608	0.057877	-0.062	0.950
Previous RT	0.070863	0.013922	5.090	<0.001
Contrast*Previous RT	-0.104218	0.019003	-5.484	<0.001
CT*Previous RT	0.091393	0.019255	4.747	<0.001
Contrast*CT*Previous RT	0.063083	0.027095	2.328	0.020

We now consider the effects involving previous RT (shaded cells in **Table 2**). There was a main effect of previous RT, indicating that previous RT and current trial RT were correlated. Critically, we also observed that previous RT and contrast interacted. This indicates that contrast effects were larger the faster the previous RT. Previous RT also interacted with context transition, indicating a higher correlation between previous RT and current trial RT when the context repeated. Particularly interesting, previous RT, contrast, and context transition interacted. This indicates that the effect of previous RT on the contrast effect was larger when the context alternated. Overall, then, the results fit perfectly with the predictions of the temporal learning account.

## DISCUSSION

The current paper makes two novel contributions to the literature. First, the results of our experiment are consistent with our suggestion that temporal learning can function in a context-specific manner. Specifically, the contrast effect was found to be larger in the mostly easy location context relative to the mostly hard context. Thus, the magnitude of an effect on the current trial is influenced not only by previous trial RTs, but also by contextual cues. Interestingly, this context-specific proportion easy effect was not modulated by the contrast of the previous trial or by whether or not the context (i.e., location) repeated. The lack of an effect for location repetitions is particularly interesting, because it shows that the appearance of the context-specific proportion easy effect is not solely driven by those trials on which the context repeats. This would seem to suggest that participants have two learned rhythms, one for each location context, that they can flexibly switch between depending on the location in which the stimulus appears. Note that previous contrast did impact the magnitude of the current trial contrast effect, but this effect was eliminated when previous RT was included as a factor. This suggests that previous trial RTs influence the size of the contrast effect, but not previous contrast (which is correlated with previous RT). This is consistent with results from analyses on list-level effects, where the same pattern of results was observed (Schmidt, 2013b).

The mixed model analyses including previous RT were particularly interesting. In line with predictions from the temporal learning account, previous RT not only correlated with current trial RT, but also affected the size of the contrast effect. As predicted, the contrast effect increased as previous RT sped up. Moreover, this effect of previous RT on the contrast effect was found to be larger when the context (i.e., location) repeated. This is consistent with the notion that participants learn a rhythm for each location, because the previous RT on a context repetition belongs to the same context as the current trial, whereas on a context alternation it belongs to a different context.

The second novel contribution of the current work is that our results hint at an alternative explanation of the CSPC effect. While previous accounts might have attributed such findings to hemisphere-specific processing (Corballis and Gratton, 2003) or context-specific adjustments of cognitive control (Crump et al., 2006; Bugg et al., 2008; Wendt et al., 2008; Crump and Milliken, 2009), the present results suggest that such effects might instead be explainable by context-specific temporal learning. Indeed, the context-specific conflict adaptation account argues that the CSPC effect is driven by attentional adjustments to the differing rates of conflict in each location. If the CSPC effect is argued to be solely explainable by such attentional adjustments, then the current observation of a context-specific proportion easy effect should not have been predicted. Given that the present contrast design used no conflicting stimuli (indeed, there were no distracters in the task), an interaction between proportion easy and contrast should not have occurred. Furthermore, it is not clear how any such attentional filtering account could explain the influences of previous RT that we observed in our data, at least not without considerable added assumptions. Of course, this does not mean that conflict adaptation does not play a role in the CSPC effect, but our results might suggest, at minimum, that the contribution of conflict adaptation to such an effect is probably overestimated due to temporal learning biases.

Unlike attentional accounts, the simple temporal learning account has no difficulties with the current findings. The effect of context on the magnitude of difficulty effects (e.g., congruency or contrast) is proposed, according to such an account, to be unrelated to conflict. Thus, context-specific effects should not be eliminated by removing conflict from the task. The temporal learning view only needs to assume that participants can learn different temporal rhythms for two contexts. If they are in a faster rhythm (i.e., have earlier temporal expectancies) in the mostly easy context (i.e., because of the large number of high contrast or congruent trials), then they will have a larger effect relative to the slow-rhythm (i.e., later temporal expectancies) mostly hard context.

Another interesting result that might be seen as consistent with the temporal learning view comes from Wendt and Kiesel (2011). They presented participants with a CSPC task in which *foreperiod* (i.e., the time between fixation and stimulus presentation) was the contextual cue. Specifically, a short foreperiod (200 ms) was associated with mostly congruent stimuli and a long foreperiod (1200 ms) was associated with mostly incongruent stimuli, or vice versa. Congruency effects were larger with the mostly congruent foreperiod. This is an interesting finding, as some form of timing process is necessary to explain such results. While it can certainly be argued that attentional filtering might be modulated over time with contextual cues (though this would require some changes in thinking about conflict adaptation effects), such results could alternatively be argued to be due to participants learning when to respond based on stimulus onset. The idea that participants can learn about differing temporal intervals is already an inherent part of the temporal learning account, meaning that such results fit quite nicely with the temporal learning view without any necessary adjustments to the account. As pointed out by Wendt and Kiesel, this is not the case for extant models of conflict monitoring (e.g., Blais et al., 2007; Verguts and Notebaert, 2008), which would need some retuning to allow for such time-based effects. Indeed, one of such changes would have to be a mechanism to learn about timing, further indicating that temporal learning of one form or another is a highly plausible mechanism for producing CSPC effects.

As discussed in the Introduction, the temporal learning account could potentially also explain the transfer effects observed by Crump and Milliken (2009; see also, Heinemann et al., 2009; Reuss et al., 2014) for contingency-unbiased items. Indeed, the items in the current report were entirely contingency-unbiased. That is, there were no distracting stimuli that could provide a predictive cue for the likely response. Similarly, the location context was completely un-predictive of what response would follow. Of course, some letter–contrast–location combinations were more frequent than others, but the previously discussed results of Schmidt (2014) demonstrate that proportion easy effects with contrast are not (at least primarily) item-specific. Future work might aim at testing context and transfer items in a context-specific proportion easy task to add further credence to this notion.

The present research is not without limitations, however. Note that the present results do not rule out the possibility that conflict adaptation also contributes to the CSPC effect. The conflict adaptation account does not specifically predict that a context-specific proportion easy effect should *not* occur. It merely does not predict such an effect. It could be that both conflict adaptation and temporal learning play a role in the CSPC effect. Of course, this is a less parsimonious account than suggesting that both the context-specific *PC* and context-specific *proportion easy* effects are explainable by the same (e.g., temporal learning) mechanism. Moreover, even if context-specific conflict adaptation *does* occur, the present results would suggest that the CSPC effect is likely to be confounded with temporal learning biases. Thus, the best possible outcome that remains for the conflict adaptation view is probably the conclusion that the CSPC effect overestimates the contribution of conflict adaptation processes. Future research might aim to attempt to dissociate the separate influences of temporal learning and conflict adaptation on the CSPC effect. As already pointed out, this is unfortunately a dissociation that will be difficult if not impossible to produce, given how inherently confounded speed-of-responding and congruency are. It is certainly our hope, however, that the current work might serve to inspire other researchers to find a solution to this predicament.

As another caveat, there were a few differences between the context-specific proportion easy data observed here and data from CSPC experiments. For instance, a main effect of proportion easy was observed, such that responses were overall slower in the mostly easy condition. More specifically, this main effect seemed to be the result of the context-specific proportion easy effect being driven exclusively by hard items. However, in CSPC experiments it is uncertain whether such a main effect is observed. In King et al. (2012a; see also King et al., 2012b) the CSPC effect was driven by seemingly symmetric effects on congruent and incongruent trials. On the other hand, the exact same pattern of interference-driven effects that we observed *was* observed in CSPC error rates by these authors. Crump et al. (2006) do not report tests for main effects, but their Experiment 1 RTs and Experiment 2a error rates appear numerically consistent with our findings. Their Experiment 2a RT data suggest the reverse pattern, however, with the CSPC seemingly driven by congruent items. As pointed out by Schmidt (2014), proportion congruency manipulations of all types, much like proportion easy effects observed in our lab, seem to provide quite inconsistent patterns regarding whether the effect is located in the congruent trials, incongruent trials, or both. It is not clear how any account explains these inconsistencies, and we would suggest that future work might aim to explain not only PC interactions, but also the precise pattern of means.

One possible explanation is the presence of floor or ceiling effects. In some experiments, responses to easy (e.g., congruent) items might be fast enough that no further benefit can be gained from temporal expectancies (or some other mechanism, such as conflict adaptation). In other experiments, responses to hard (e.g., incongruent) items might be slow enough that participants are responding at a maximum slow rate, temporal expectancies or not. For instance, responding might be thresholded for such hard items. Error rates might be informative in such a case. This floor/ceiling argument, however, is admittedly post hoc and would need corroboration from actual data. Another possibility might be overall differences in response caution for differing contexts, though this would seem at first glance to predict the reverse main effect of context observed in the current report. On the other hand, an early temporal learning account by Grice (1968) suggested that in an overall harder context, the threshold for responding might be reduced to expedite processing of difficult items. This notion *is* consistent with the finding of slower responses for both the easy and hard item types in the mostly easy condition. Whatever the explanation, note that the observed main effect does not present an inherent problem for the temporal learning account, as the overall mean RT of responses in mostly easy context was faster than in the mostly hard context.^2^

As another limitation, it is noteworthy that we did not find a modulation of the context-specific proportion easy effect as a function of whether or not the context repeated from one trial to the next. Such modulations have been observed in CSPC experiments (King et al., 2012a,b). Though not impossible that we simply lacked the statistical power to detect such a modulation, no clear evidence for one was observed. This inconsistency does leave open the possibility that the context-specific proportion easy effect is not driven by the same mechanism as the CSPC effect, which would undermine the temporal learning account of CSPC effects suggested in the current manuscript. On the other hand, we provided an important control on the context transition analysis by including previous contrast in the analysis. This is important, because previous trial contrast is highly confounded with context transition. Unfortunately, the same control was not used in previous research investigating the role of context transition in CSPC experiments. Specifically, previous trial congruency was not coded along with context transition. Thus, the observation of larger CSPC effects following context repetitions relative to context alternations could have been due to a previous trial congruency confound. Indeed, the direction of observed effects is consistent with such a confound. Furthermore, such interactions between previous and current trial congruency are well documented (Gratton et al., 1992) and may be, in full or in part, driven by feature repetition or other learning biases (Mayr et al., 2003; Hommel et al., 2004; Schmidt and De Houwer, 2011; Mordkoff, 2012). Thus, there is strong reason to suspect a confound in the analyses of King and colleagues. As such, future research or reanalysis of existing data to answer this question is well warranted.

As another limitation, while our experiment was designed to test the *a priori* hypothesis that a context-specific proportion easy effect could be observed in the absence of a conflict manipulation due to a temporal learning process, it is alternatively possible that yet another account explains either the context-specific proportion easy effect or *both* the context-specific proportion easy and CSPC effects. One account, intimately related to the temporal learning account presented here, is the response caution account. According the response caution account, context-specific effects could be driven by increases in the response threshold when experiencing unexpected stimulus combinations for a context. For instance, a low contrast item is not expected in the mostly easy context, and this might lead to an increase in the response threshold, thus delaying responding. A similar process would occur for high contrast items in the mostly hard context.

Indeed, evidence for a response threshold account of CSPC effects has been presented by King et al. (2012a). They used a quantitative model to test how well a threshold account fit the data relative to an evidence accrual model (the latter of which is consistent with a conflict adaptation process). The response threshold model was found to provide a much better fit than the evidence accrual model. The authors therefore argued that the CSPC effect is better explained by response caution than conflict adaptation. It should be noted that the response caution predictions match those of the temporal learning perspective, because both accounts predict a relatively lower threshold for expected stimuli (congruent mostly congruent and incongruent mostly incongruent) relative to unexpected stimuli (congruent mostly incongruent and incongruent mostly congruent). Similar time-based accounts, such as the adaptation to the statistics of the environment (ASE) model (e.g., Kinoshita et al., 2011), are also consistent with such results. We therefore think that determining which variant of these response threshold models provides the best fit to proportion easy and PC effects is an important goal for future research to address.

Though inconsistent with the above-mentioned finding that CSPC effects are more in line with a response threshold rather than evidence accrual mechanism, it could nevertheless be proposed that some other form of attentional filtering mechanism explains the context-specific proportion easy effect observed in the current manuscript. While it seems that conflict adaptation can be safely ruled out for the context-specific proportion easy effects observed with the present design, perhaps it could be argued that the cognitive system learns to better extract target information of the most frequent contrast level in each context. That is, high contrast stimuli might be better processed in the mostly easy location, and low contrast stimuli in the mostly hard location via some form of attentional capture (e.g., see Cosman and Vecera, 2014; Thomson et al., 2014) of the most likely contrast level. It is not entirely clear how such an attentional mechanism would work, however. For instance, it seems unlikely that information accrual of low contrast items in the mostly hard context would be improved without similarly improving high contrast items in the same context. The plausibility of this account is further weakened by the fact that the modeling results of King et al. (2012a) seem to argue against an evidence accrual account of context-specific effects. Further still, it is not clear how such an account would explain the effects of previous RT we observed. Still, it should be acknowledged that the manipulation of “easy” and “hard” did involve using stimuli of differing luminance. This is not optimal for comparison with CSPC experiments, where luminance is equated across easy (congruent) and hard (incongruent) items. Future experiments with different manipulations of stimulus ease would thus be a welcome addition.

It should also be noted that the temporal learning view does share some similarities with the conflict adaptation view. In both accounts, it is assumed that contextual information is used to adjust performance. In the conflict adaptation view, attention is adjusted to minimize conflict. In the temporal learning view, expectancies are adjusted to maximize the speed of responding. Both accounts therefore propose an adjustment of performance in order to benefit the task goal, but merely differ in *what* is adapted to (i.e., conflict versus temporal information) and *how* performance is adjusted (i.e., attentional modulations versus temporal expectancies). These are not trivial differences, of course, and we hope that future research will further the investigation of these issues. The current results only provide a proof-of-principle that temporal learning might provide a sufficient explanation of CSPC effects. Further work will be required to draw more definitive conclusions.

At the broader level, the current results suggest that temporal learning occurs in a context-specific fashion and that switching between contexts can occur on a relatively quick, trial-by-trial basis. This is an interesting finding in its own respect that might be investigated further in future research. Rapid context-specificity in temporal learning also need not be viewed as unintuitive. While many rhythmic behaviors may entail producing an action in equally spaced intervals, this is not always the case. For instance, not all notes in a song will be quarter notes. Some notes will come sooner or later, often with deliberate syncopation. Thus, even with the most obvious example of rhythmic behavior (i.e., music), context (in this case, the notes that came before the current one) plays an important role in modifying behavior.

## Conflict of Interest Statement

The authors declare that the research was conducted in the absence of any commercial or financial relationships that could be construed as a potential conflict of interest.

## Footnotes

1 In this experiment, half of the participants received mostly easy stimuli above fixation, and mostly hard stimuli below fixation. The other half of the participants had the reverse. Thus, both proportion easy and stimulus location were manipulated within-groups, but the *interaction* between proportion easy and stimulus location was manipulated between-groups. Standard repeated-measures ANOVA has no easy way of handling such data, whereas LME modeling does. Note that the counterbalancing factor could be used in place of location in a repeated-measures ANOVA. As we confirmed, such an analysis produces exactly the same results as the LME, but the location effects are represented by very unintuitive interactions between counterbalancing order and proportion easy.

2 An anonymous reviewer pointed out that the main effect of proportion easy might seem problematic for the temporal learning account. Specifically, this main effect seems to indicate that RTs were slower in the *mostly easy* context than in the mostly hard context. This might seem inconsistent with the temporal learning account, which requires the reverse pattern (i.e., *faster* responses in the mostly easy context). However, note that this main effect is computed by averaging the mean RT for high contrast with the mean RT for low contrast in each context. Thus, such an analysis ignores the relative *frequency* of high and low contrast items in each context. Because there are much more high contrast items in the mostly easy context than the mostly hard context, the average overall RT for the mostly easy location is actually faster. That is, there are many high contrast trials and few low contrast trials in the mostly easy context, meaning that most of the responses in the mostly easy context are fast. In the mostly hard context, it is the reverse, with a large number of (slow) low contrast items. Indeed, if one simply computes the average RT for each context (i.e., ignoring the distinction between high and low contrast items), overall RTs were significantly faster in the mostly easy context (659 ms) than the mostly hard context (693 ms), *F*_(1,16323)_ = 87.283, *p* < .001, $\eta_{p}^{2}$ = 0.75. This 34 ms difference therefore shows that the temporal regularity did indeed exist for learning context-specific regularities.

3 Note that with regard to the statistics that follow the –1000/RT inverse transform is equivalent to a 1/RT inverse transform. The only differences are that the negative sign preserves the more intuitive direction of effects, and the 1000 multiplier reduces the numbers of decimal places in the parameter estimates.
